# Typhoid Fever

**Published:** 1860-08

**Authors:** V. H. Taliaferro

**Affiliations:** Columbus, Georgia


VOL. V.] AUGUST, 1860. [NO. 12.

### ORIGINAL COMMUNICATIONS.

### ARTICLE I.

*Typhoid Fever;* By V. H. TALIAFERRO, M. D., Columbus, Georgia.

No disease, perhaps, has been so extensively discussed through the Medical press as Typhoid Fever, and we must, therefore, beg of the profession a little patience for what may seem to be an unpardonable assumption on our part in the advancement of a *new theory*, by which we confidently hope and expect to throw some light, at least, upon the clouded and unsettled pathology of this disease. The confused state of the professional mind as to its *real nature*, and the unsatisfactory result of the various modes of treatment as set forth by Medical authors, we claim as sufficient extenuation for what may seem to be an unnecessary expenditure of time and words upon a subject so long and ably discussed by some of the greatest minds in the medical profession. If the treatment of disease proves unsatisfactory, the conclusion to which we reasonably and naturally arrive, is that the true pathology has been mistaken. We should, therefore, make increasing and diligent investigations until the *truth* has been found, for it is only when the real seat and nature of disease is fully and clearly comprehended, that the treatment becomes an easy and a simple matter.

It was at one time claimed that Typhoid Fever was produced only by a collection and confinement of a number of persons together in close apartments, and was accordingly termed Jail or Ship Fever, but since the discovery of the lesion of peyers glands, which, ordinarily, but not invariably, accompanies the disease, it has been popularly styled enteric, &c.; and by very many, some of whom we know to be distinguished, this lesion is regarded as a full and complete explanation of the curious phenomena presented in Typhoid Fever. Such conclusions we can only regard as erroneous, for several reasons: 1st, These glands are not invariably involved, as positively revealed and established by examinations post mortem. 2nd, The severity of the symptoms seem not to be governed by the extent of the ulcerations, as we sometimes find that when the abdominal symptoms have been most intense, the glands present but slight disease, and in some cases none whatever. 3rd, We cannot explain by this glandular ulceration the great nervous and muscular prostration. 4th, We would expect *inflammation* to precede ulceration of these glands, and Typhoid Fever consequently ushered in with *acute inflammatory symptoms*, as in the phlegmasia, which is far from being the case.

Again, the medulla oblongata is strongly and ably claimed as the primary seat of this disease, and though we are unable fully to adopt the theory, we can certainly explain by it many of the most alarming symptoms of Typhoid Fever; and it is doubtless implicated, but we must regard it as a *secondary* result. That the nervous centres are unusually and powerfully affected there can be no question. Great and oftentimes alarming prostration is the first evidence of an attack of Typhoid Fever—all the symptoms denoting, conclusively, that some overwhelming impression has been made upon the nervous centres, and that impression the result of some intense and powerful poison introduced into the blood. Watson, a long established and distinguished author, cites us, as the exciting cause of the disease, to the introduction into the blood of a 'specific poison,' but is at a loss to know the character and nature of that poison. Now we beg leave to claim that this poison arises from a disturbance of the *cutaneous functions, whereby the perspiratory fluids are in part retained in the circulating mass*, poisoning and contaminating the whole, and paralyzing, as it were, the performance of the normal functions of the nervous centres, by the intensity of its action.

It will facilitate our purpose to notice here, very briefly, the physiology of the skin, and the character of its secretions. More than twelve months since we published an article upon *the skin*, in which we endeavored to bring prominently before the profession the importance of its great functions, and the wonderful influence it may be made to exercise in the successful management of disease. We propose now to make a few extracts from that article. In the cutaneous surface we have presented to us the most extensive organ in the system, and one which eliminates, ordinarily, a larger amount than either the kidneys, lungs, liver, or intestinal canal. And as a proof that the matter thus eliminated is equally obnoxious with that of other secretions, when retained in the blood, we have only to remember that a suppression of the skins functions, by the application of impervious substances to the surface, produces death in an exceedingly short time. According to Erasmus Wilson, "The number of square inches of surface in a man of ordinary heighth and bulk, is 2,500; the number of pores thereof, 7,000,000; the number of inches of perspiratory tube, 1,750,000, that is 175,833 feet, or nearly 28 miles." From these twenty-eight miles of perspiratory tubules there escapes every twenty-four hours from two to three pounds of insensible perspiration, the chemical constituents of which are water, nitrogen, animal extract, fat, carbonic acid, with its salts, carbonates of soda and lime, lactic acid, with its compounds, lactate of ammonia, acetic acid, butyric acid, chloride of sodium, hydrochlorate of ammonia, phosphates of soda and lime, sulphate of soda, salts of potash, and protoxide of iron. From two to three pounds daily, and sometimes double that amount of these materials are thrown from the blood upon the surface, as unfit for further use in the economy, and consequently deleterious to its well being. If permitted to collect from day to day, and, as in very many cases, from month to month, upon the surface, the delicate mouths of the perspiratory vessels become clogged, and consequently disabled in their important functions of cleansing and purifying the circulating mass, which is in consequence forced to retain offensive elements which contaminate and change its quality, and paralyze the normal tone and energy of the nervous forces. So intensely poisonous are the perspiratory fluids when prepared for elimination from the system, we cannot presume otherwise than their retention in the blood would necessarily produce disease of an aggravated and serious character.

Not only does the skin perform this important office of elimination, but acts in the capacity of a great regulator of the animal heat. It becomes, also, from the extensive capillary net work so thickly and beautifully inlaid beneath the epidermis, an important adjunct to the lungs in aeration of the blood. The sensory nerves, too, which pierce the papillæ of the true skin, would seem as so many watchful sentinels to guard the life-giving functions within, and to note every impression however delicate. We readily perceive, then, that any obstruction to the free action of the perspiratory vessels, not only poisons the blood, but deranges the arterializing function of the skin, and obtunds its natural and delicate sensibilities, and as a natural consequence undue excitation must fall upon distant organs, as the lung, intestinal canal, &c., which we find act vicariously with the skin, whenever its extensive tubing apparatus is disordered, as evidenced by the diarrhea and bronchial irritations which follow. These organs, as well as the kidneys, hold a close relationship to the cutaneous surface and act in sympathy with its disorders—organs, it must be remembered, which present in Typhoid Fever serious and fearful complications, except, perhaps, the kidneys, which, however, we have frequently seen excessive in their action, and which would seem to be influenced by the quality and quantity of the intestinal discharges. In this disease the kidneys certainly act, as a generality, more profusely than in other fevers, and as before remarked, sometimes excessively, the explanation of which, previous to the adoption of our theory, we were wholly unable to explain.

Those who have had experience in Typhoid Fever must have been struck with the very usual bronchial or lung complication, and instead of presenting the strange combination of a sthenic and asthenic disease, so beautifully portrayed by Dickson, is but a natural effect arising from the true seat of the disease—the skin. The lungs have no special sympathy with Peyers glands, or the medulla oblongata, neither are they directly disturbed by malarial and other atmospheric poisons, but upon the principle of cutaneous obstruction an explanation is easily removed. No organs hold so close communion as does the skin and the pulmonary organs.

It will of course be asked how we are to explain upon our principle, the lesion in Typhoid Fever of the glands of Peyer. In the first place, disturbance of the cutaneous functions is felt more or less in the whole system of mucus and subcutaneous glands, evidences of which are clearly presented in the irritation and enlargement of the tonsils, lachrymal and sub-maxillary glands, from changes of temperature and exposure. Thus it appears that cutaneous disturbance predisposes as well as produces irritation in the mucus and subcutaneous glands, and as Peyers glands lie immediately within the tract of the small intestines, themselves the seat of serious disorder, we cannot well see how they could escape more or less implication. Thus are we enabled to explain the phenomena in Peyers glands so eagerly sought for by the scalpel in this disease, and likewise do we explain the very common disturbance of the sub-maxillary glands, which of themselves present not unfrequently serious impediment to successful results.

The persistent and aggravated diarrhea of Typhoid Fever affords one of the strongest proofs to the establishment of our principle, for every physician acquainted with the physiology of the skin knows how quickly and surely a disturbance of its functions makes an impress upon the mucus lining of the intestinal tract. No argument is necessary to establish this point.

In the whole category of fevers, none are ushered in with the same amount of prostration as Typhoid Fever, and later in the disease the unusual and oftentimes alarming depression, denotes evidently that the nervous forces have been most powerfully overcome by the action of some "specific poison." Watson says: "But the exciting cause in most cases, probably in all, is a *specific poison* [Italics ours] received into the blood. The first evidence of the circulation of this altered fluid, is depression of the powers and functions of animal life." From whence came this "specific poison" we are not told. By reference to the chemical constituents of the perspiratory fluid, given in a preceding page, it will be found to contain elements fully capable of producing all the alarming and hazardous effects upon the "powers and functions of animal life" presented in Typhoid Fever. There can be no inconsistency in referring this poison, of which Watson speaks, to the cutaneous fluids, particularly as we find the skin almost without an exception, *uncleanly* and *offensive*, emitting a peculiar fœtid odor—the perspiratory vessels disabled in their functions and forcing upon the blood a most intense and powerful poison.

In Typhoid Fever, we have a decided tendency to disintegration and putrescence of the tissues, evidences of which are strikingly presented in the facture of the body, the dry, black and incrusted tongue, dark sordes upon the teeth, and occasional mortification and sloughing of the inferior extremities. Here, then, is just such a condition as we would very reasonably expect from a retention in the blood of the perspiratory matter. We are unprepared to say what particular change the blood undergoes, from the action of the perspiratory fluids; but we would certainly look for a decided change or disorganization of its elements, and we accordingly find that in Typhoid Fever, the blood has so changed as to loose very remarkably its powers of coagulation. It is by no means strange or unnatural that carbonic acid and its salts, animal extract, &c., chemically combined, as in the perspiratory fluids, should produce material changes in the blood, when unduly retained, not only lessening or destroying its coagulating powers, but establishing a decided tendency to putrescence, which becomes thoroughly impressed by the circulating mass, upon all the tissues of the body.

Again, experience has well testified that the usual mode of death in typhoid fever is by coma, and repeated and continued investigations *post mortem*, have clearly established the fact that this coma is the result of no lesion of the brain or its membranes. Now there is but one other mode by which by which coma may be produced, and that is the circulation in the blood of a "specific poison," as *carbonic acid*, opium, animal poisons, &c.—such poisons, indeed, as supplied to the blood in typhoid fever, by obstructions and derangements in the cutaneous functions.

We are through with our theory, and if objections are found to it which can be sustained, we would be glad to know them through the medical press. This subject affords an important and interesting field for investigation; and if our principles are truly founded upon a *false basis*, we shall not mourn to see them exploded. We should have been glad to have been more elaborate in the elucidation of our subject, *but* for fear of tedious length to both editors and readers.

We will now consider in as condensed a manner as it is possible for us to do, the treatment of Typhoid Fever, the success of which we may remark, first led us into the light of the principles we have adopted. In the management of this disease, our remedies should be directed to the fulfilment of two important indications, viz: the deranged condition of the cutaneous functions, and its consequent effects upon the system, embracing more particularly the nervous and circulatory systems, and the intestinal canal with its mucus glandulæ.

When first called to a patient with this disease, the skin will be found incrusted, as it were, by the collection of the perspiratory fluid, and imparting to the hand a dry harsh and unpleasant feel. The first thing, therefore, should be a thorough cleansing with tepid water and soap, with a *coarse towel*, which is better than sponge, from the fact of the greater friction applied in its use. This should be repeated early *every morning*, and the patient will invariably be left in the enjoyment of the most pleasant and refreshing sensations. At each ablution the patient's clothing, as also his bedding, its linen, &c., should all be changed. In this regard we would recommend the strictest attention, from the fact that not only are the cutaneous vessels diseased, but the emanations from the body are from a *diseased blood*, which would of course collect from day to day in the clothing, bedding, &c., filling the room with noxious and putrid odors, and be more or less reabsorbed and rebreathed, embarrassing, of course, very greatly a favorable termination of the disease. Every thing, therefore, about and around the patient should be kept, in every sense of the word, cleanly—perfectly free from any collection of effete materials thrown off by the cutaneous vessels.

In addition to the above, the patient should, every night, have his *entire surface* well "greased," using as much friction in its application as comfort will admit. For this purpose, either lard, olive oil, or *bacon rind* may be used. If the lard or oil are used, they should contain as much *salt* as they can be made to dissolve. The reasons for annointing our Typhoid patients, are simply these: In the first place, we render *soft* and *pliant* a hard, dry skin, loosening and opening its texture, thereby promoting its life-sustaining functions, and relieving the nervous system of the irritation imparted to it by a dry and unyielding cuticle to the sensory nerves which so thickly and beautifully beset the true skin. Here then is really the true secret of the calming and soothing influences obtained by anointing the surface. The sensory nerves which shoot out from the great centers piercing the numberless papillae of the true skin, and like so many electric wires, telegraphs to the centers the impression they have received, takes cognizance of every passing breeze, its force and temperature, its purity, dryness or dampness, all being duly conveyed to and chronicled upon the great and wonder working brain.

These impressions, though within themselves, of little importance, when made upon the whole sensory system, become more or less powerful in their effects.

Take for example, one naked arm exposed for a short time to a cold atmosphere, and no observable effects are produced upon the general system, but if the entire cutaneous surface be exposed, serious results may ensue. Again, a slight burn produces but little constitutional disturbance, but if extensive will greatly hazard, or it may be destroy life. Now, upon the very same principle will a *dry*, incrusted state of the skin effect an irritable condition of the nervous system, and likewise the application of oils, which soften the skin, imparts a calming soothing influence.

The salt is added for its purifying and disinfectant virtues, which it possesses to a very great degree, and therefore exerts a highly salutary and counter influence upon the altered and putrescent condition of the blood, and indeed of all the tissues. Not only are its benefits obtained by the absorbent powers of the skin which carries it directly into the circulating mass, but, by its neutralizing powers upon the fœtid odors of the body. He who adopts this greasing practice, whether empirically or upon principle, will have sufficient reasons from its good results, to continue it so long as he can find a case in which to apply it. We speak from no limited observation, but from experience obtained in the malignant epidemics which have been spreading over the middle sections of Georgia since 1850.

Another important advantage obtained in the bathing and greasing of our Typhoid patients, is the *exercise* given them in the operation. Now this will doubtless appear to many an extravagant idea, but when we come to consider that Typhoid patients will, in the latter stages of the disease, lie upon their backs in the same position for days and weeks, if permitted, favoring congestion in the languid and almost lifeless viscera, encouraging the debility of the nervous system and torpidity of all the functions of life, the little exercise given to the entire system by *rubbing the surface* cannot possibly act otherwise in this disease than beneficial, by imparting a healthful tone to the sinking powers of life.

The feeding of Typhoid patients should demand the closest attention. From our own observation, corroborated by that of others, we cannot believe otherwise than that hundreds die in this disease from the want of sufficient nourishment. It is a disease which must be fed, and richly fed, or else the rapid disintegration which characterize it will soon consume the life sustaining elements which feeds and supports all the functions and forces of life, and which can only be supplied and renewed from nutrient materials introduced into the system. Therefore, regularly every two or three hours, as the case may seem to demand, wine whey, milk punch, or beef tea, should be administered in small quantities. This dietetic treatment early commenced, will save the lives of many, and invariably establish a more rapid and effectual convalescence.

Air and sunlight should both be admitted freely into the chambers of Typhoid subjects—the one to establish a perfect æration of the blood, and the other for its mildly stimulant and tonic influences to the nervous and circulatory systems. Impoverishment of the blood cannot be more effectually produced than by living where the sun's light is extended. We would therefore recommend that these salutary agents of nature flow in continuous streams through the apartments of Typhoid patients.

Thus it will be seen that bathing, anointing, diet, air and sunlight, constitute our hygienic treatment, upon which indeed we place our great reliance. And here we would remark, that we look forward to the day with pleasing hopes, when the great laws of hygiene will be properly appreciated and applied to the establishment of the normal function of the economy.

Our *medical* treatment is told in a few words. We commence at once, when called to the disease, whether in its primary or latter stages, the administration of Chlorate of Potassa in saturated solution, with the view to its disinfectant virtues, its tonic and alterative influences to the blood, and its controlling powers over ulcerated and inflamed mucus surfaces. Our habit is to combine with it Tinct. Virat. Viride as a sedative to the circulation in the following proportion:

Chloras Potas, sat, sol ℥ viij

Tinct Virat Viride ʒ j.

Of this mixture, one table spoonfull to an adult every three or four hours. If there is great restlessness, we combine with this prescription ʒ j of Tilsens fluid extract of aconite.—This ℞. should be given through the day, and at night a single dose of Opium, and the patient left undisturbed until the next morning. Wine or brandy should be given as the case seems to demand.

Some two years ago we published an article in the Atlanta Medical & Surgical Journal upon the use of Chlorate of Potassa in Typhoid Fever, and we have been highly gratified to find that since that time it has been adopted by the profession in different parts of the United States, and the above ℞. copied into several recent publications, among which we may mention an elaborate and highly valuable treatise upon Enteric Fever, by James E. Reeves, M. D., Philadelphia.

